# Clinical Presentation and Surgical Management of a Grynfelt Hernia: Report of a Clinical Case and Literature Review

**DOI:** 10.1155/cris/5634242

**Published:** 2025-03-27

**Authors:** Pabel Ruben Carbajal Cabrera, Ruben Daniel Pérez López, Yunuen Ailyn Morales Tercero, Itzel Ocampo Barrero

**Affiliations:** ^1^Department of General Surgery, Universidad Nacional Autónoma de México, Mexico City, Mexico; ^2^Department of General Surgery, Institute for Social Security and Services for State Workers, Mexico City, Mexico

## Abstract

**Background:** Grynfelt's lumbar hernia is the rarest of all abdominal wall hernias, accounting for between 1.5% and 2% of cases, with only 300–350 instances described to date. Lumbar hernias can be congenital or acquired, often triggered by trauma or surgery (iatrogenic). Diagnosis is clinical and confirmed via computed tomography. Surgical intervention is required for resolution, with repair performed either through open or laparoscopic surgery.

**Material and Methods:** We present the case of a young female with no prior surgical or traumatic history, in whom the diagnosis of Grynfelt's hernia was made.

**Results:** The patient underwent elective left lumbotomy surgery with hernioplasty using a supra-aponeurotic polypropylene mesh. Postsurgical recovery was adequate, and she was discharged 4 h after surgery. Follow-up in the general surgery outpatient clinic occurred at 20 days, 1, 3, and 6 months, with no recurrence, complications, or incidents.

**Conclusion:** Grynfelt's hernia is a rare entity that requires a high index of suspicion for accurate diagnosis. Although cases are often asymptomatic, untreated hernias can lead to significant morbidity. Early recognition and timely surgical intervention are crucial for symptom relief and prevention of complications. In this case report, surgical management involved hernioplasty through a left lumbotomy approach, repairing the hernia defect and reducing the hernia content. Supra-aponeurotic mesh was placed to ensure adequate closure. Given the rarity of this pathology, no specific management guidelines exist in the literature. Therefore, the decision for this type of repair was based on intraoperative findings. Further research is needed to clarify management strategies and optimize outcomes for patients with Grynfelt's hernia.

## 1. Introduction

A lumbar hernia is the protrusion of intraperitoneal or extraperitoneal contents through a defect in the posterolateral abdominal wall. These hernias are classified based on the anatomical triangle affected, first described in 1783 and 1866, respectively, and are named after the Jean–Louis Petit triangle (lower) [1] and the Grynfeltt–Lesshaft triangle (upper) [2].

With ~300–350 cases published in the literature, lumbar hernias represent 1.5%–2% of all abdominal hernias, with Grynfelt's hernia being more frequent [3]. These hernias are classified as either congenital or acquired. Approximately 20% are congenital, mainly due to defects in embryonic development, while 80% are acquired [4].

The Grynfeltt–Lesshaft triangle is an inverted triangle with boundaries formed superiorly by the twelfth thoracic rib, medially by the erector spinae and quadratus lumborum muscle group, and laterally by the internal oblique muscle [5]. The floor of this triangle is formed by the aponeurosis of the transversus muscle, and the roof is formed by the Latissimus Dorsi muscle and external oblique muscle [5].

Grynfeltt's triangle has three described weakened areas: immediately below the rib, where the transversalis fascia is not covered by the external oblique muscle; in the area where the twelfth dorsal intercostal neurovascular pedicle penetrates the fascia; and between the lower edge of the rib and Henle's ligament [6].

The aim of this article is to present a case report of a female patient with a congenital Grynfelt hernia, which has a very low prevalence. We describe the diagnostic approach and surgical treatment of Grynfelt's hernia.

## 2. Case Presentation

A 36-year-old female patient with no personal medical history. She denies smoking and has no history of other surgeries or trauma related to the lumbar region.

She presented to the Emergency Room reporting the onset of her current condition 2 years ago, characterized by intermittent stabbing pain located in the left lumbar region, associated with a left lumbar swelling that increased in size with the Valsalva maneuver. No other significant clinical symptoms were noted. On physical examination, a swelling was observed in the left posterolateral lumbar region, below the twelfth rib, measuring ~7 × 5 cm. The swelling showed no local color changes, increased in size with the Valsalva maneuver, was reducible, and soft to palpation. It was classified as a type A hernia according to the classification by Moreno-Egea et al. [7, 8].

**Figure 1:**
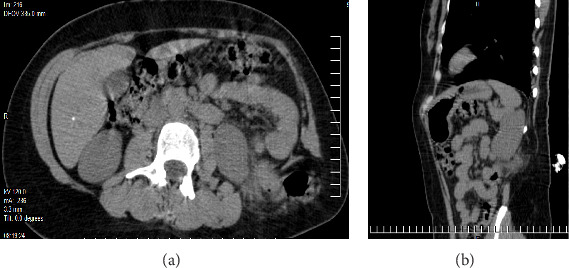
(a, b) A simple and contrast-enhanced abdominal computed tomography scan revealed an aponeurotic defect located in the left lumbar region within the Grynfeltt–Lesshaft tract, measuring 69 × 51 mm, with a hernial sac containing retroperitoneal fat.

After diagnosing and classifying the condition as a Grynfelt hernia, the patient was initially treated with NSAIDs. However, there was no clinical improvement, and further interventions were considered. A contrast-enhanced abdominal computed tomography (CT) scan revealed an aponeurotic defect located in the left lumbar region at the Grynfeltt–Lesshaft tract, measuring 69 × 51 mm, with a hernial sac containing retroperitoneal fat (Figure 1a,b).

**Figure 2:**
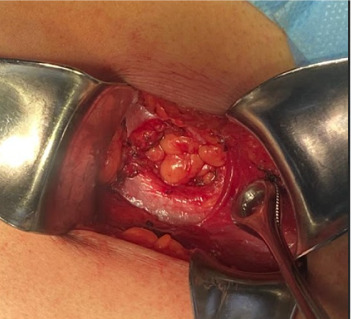
A 3 × 3 cm aponeurotic defect in the transverse abdominal muscle and a hernial sac containing retroperitoneal fat. A Foerster ring clamp is pointing at the internal oblique muscle.

**Figure 3:**
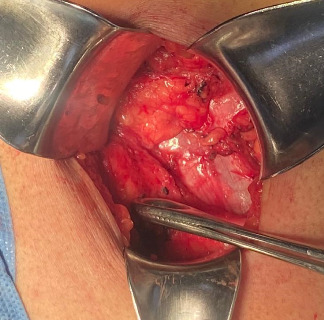
A Foerster ring clamp pointing at the latissimus dorsi muscle.

**Figure 4:**
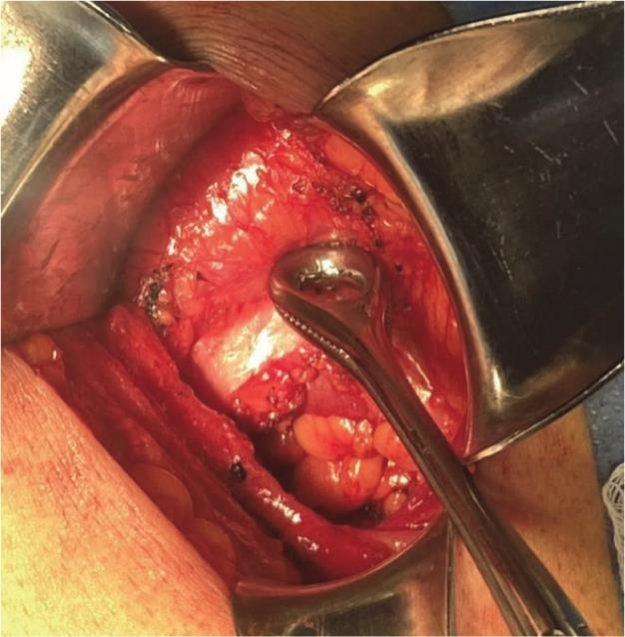
Foerster ring clamp pointing at the erector spinae muscle.

**Figure 5:**
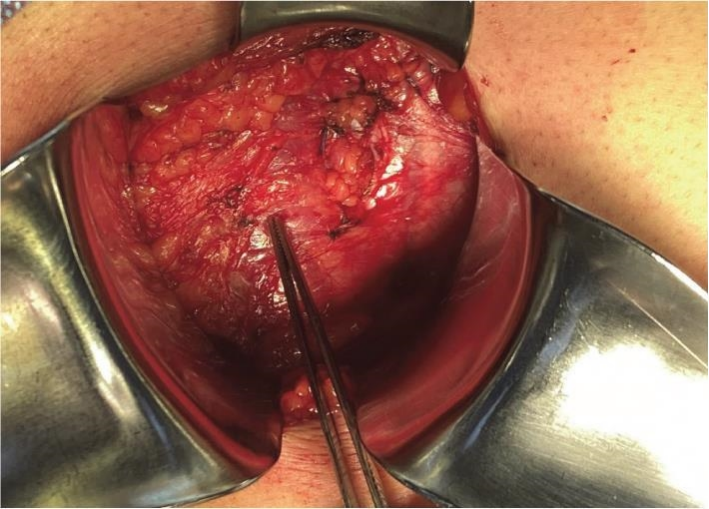
A Foerster ring clamp pointing at an aponeurotic defect in the transverse abdominal muscle, showing a 3 ×3 cm aponeurotic defect and a hernial sac containing extraperitoneal fat.

Elective surgery was scheduled. The procedure was performed under regional anesthesia. The patient was placed in the right lateral decubitus position, and asepsis of the abdominal and lumbar regions was performed using 2% chlorhexidine. A lumbotomy was performed 6 cm below the twelfth rib, with an incision ~8 cm long. The latissimus dorsi and quadratus lumborum muscles were identified, and the external oblique muscle was dissected from the internal oblique muscle (Figures 2–4). A 3 × 3 cm aponeurotic defect was found in the transverse abdominal muscle, along with a hernial sac containing retroperitoneal fat (Figure 5) [8]. Five centimeters of “healthy” aponeurosis were dissected around the periphery. The hernial sac was reduced without complications.

**Figure 6:**
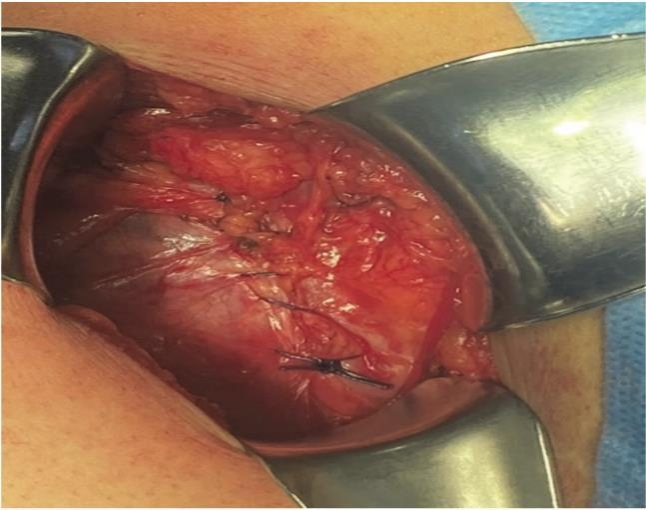
Plication of the internal oblique muscle onto the transversus abdominis muscle was performed horizontally using 1-0 polypropylene suture in single interrupted stitches.

**Figure 7:**
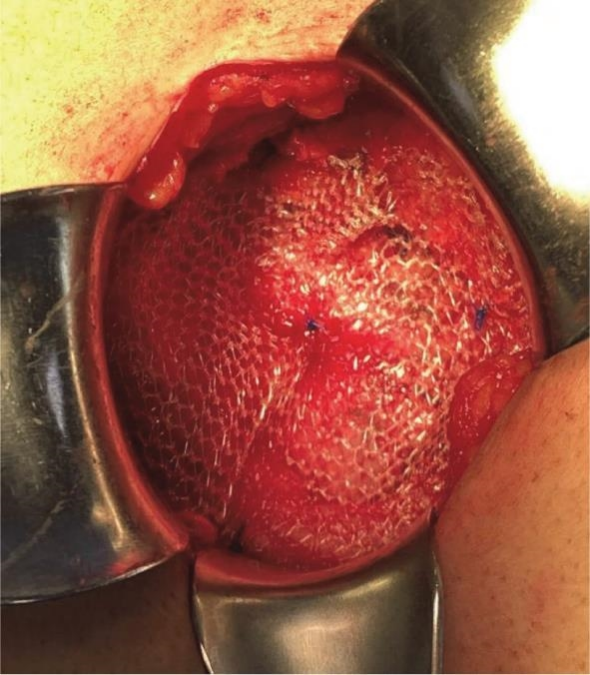
A light polypropylene mesh was applied with supraponeurotic fixation to the external oblique using 2-0 polypropylene suture.

Plication of the internal oblique muscle onto the transversus abdominis muscle was performed horizontally using 1-0 polypropylene suture in simple interrupted stitches (Figure 6). A light polypropylene mesh, measuring 8 × 8 cm, was applied and fixed supraponeurotically to the external oblique with 2-0 polypropylene suture (Figure 7). The skin was closed with 3-0 nylon, and no surgical drains were placed.

The surgery was completed in 60 min without incidents or complications. The patient had an uneventful postoperative course and was discharged 4 h after the surgery.

Follow-up was conducted in the general surgery outpatient clinic at 20 days, 1, 3, and 6 months postoperatively, with no recurrence, complications, or other incidents.

## 3. Discussion

Grynfeltt's hernias are relatively rare, occurring in the lumbar region through a defect in the superior lumbar triangle, also known as the Grynfeltt–Lesshaft triangle. According to Hafner, Wylie, and Brush [9], a general surgeon will likely only have the opportunity to repair one case of lumbar hernia in their lifetime.

Barbette first proposed the presence of these hernias in 1672, while the initial documented mention was made by de Garangeot [10] in 1731. Ravaton [11] in 1750 performed the first surgical intervention for a strangulated lumbar hernia in a pregnant woman. Petit [1] in 1783 defined the anatomical boundaries of the inferior lumbar space, whereas Grynfeltt [2] in 1866 described the superior space. In 1890, Macready documented 25 cases, including two in the superior lumbar space, coining it as the “triangle of Grynfeltt–Lesshaft.” In 1916, Goodman highlighted the prevalence of hernias in the inferior space; however, studies after 1920 indicated a higher occurrence in the superior location [8, 12]. The first laparoscopic lumbar hernia repair was reported by Burick et al. [13, 14].

Congenital presentation is rare (20%), while acquired lumbar hernias (80%) are more frequent, often resulting from incisional or traumatic origins [7]. Of the acquired defects, 55% are spontaneous or primary, and the remaining 25% are secondary [13]. Several risk factors have been described, including age (generally between 50 and 70 years), obesity, extreme thinness, cachexia, chronic wasting disease, muscle atrophy, chronic bronchitis, infected wounds, and postoperative sepsis [6]. Old age and weight loss may also contribute [15].

The diagnosis of lumbar hernia is usually based on clinical suspicion, depending on physical examination findings [16]. These hernias are often asymptomatic, though patients may sometimes report back pain, especially in the flank or lower back, and a sensation of increased bulk [7]. The size of the hernia typically increases progressively, depending on the contents, which may include retroperitoneal fat, kidney, colon, omentum, spleen, or, in very rare cases, small intestine [7]. In cases of strangulation, nausea, vomiting, and colicky pain can develop [17].

The risk of incarceration is up to 25%, with the involvement of some segment of the colon, small intestine, or omentum. The risk of strangulation ranges from 8% to 18%, depending on the size of the hernia ring. Abscess, hematoma, and neoplasia should be considered in the differential diagnosis [16].

In this case, the patient presented with intermittent stabbing pain in the left lumbar region, associated with a left lumbar mass that increased with the Valsalva maneuver, without any other symptoms. Therefore, CT was performed to rule out signs of complications.

In 97% of cases, the clinical presentation alone is not sufficient for diagnosis, particularly in very obese patients or those with small hernia sacs. In such instances, imaging studies such as abdominal ultrasound, CT, magnetic resonance imaging (MRI), and electromyography of the abdominal muscles may be necessary for diagnosis [16]. These modalities must be considered due to the rarity of this condition and the lack of a specific management protocol [18].

The only preoperative classification of Grynfelt hernias with surgical implications was proposed by Moreno-Egea [8] (Table 1). According to this classification, it is a type A hernia, and the surgical approach can be either open or laparoscopic.

Repairing Grynfelt hernias can be challenging due to their location and the potential for complications. The surgical approach remains controversial, as there are no established guidelines for the treatment of this rare condition. Options include open repair, laparoscopic repair, or even robotic-assisted repair. Each technique has its advantages and disadvantages, such as operative time, postoperative pain, hospital stay duration, and recurrence rates.

In our case, an open approach was chosen because the hospital is a secondary-level institution, and the surgeon had more experience with open management of this type of hernia. Additionally, the size of the aponeurotic defect and the presence of extraperitoneal content made this approach appropriate.

## 4. Conclusion

Grynfeltt's hernia is a rare entity that requires a high index of suspicion for diagnosis. Although most cases are asymptomatic, symptomatic cases can lead to significant morbidity if left untreated. Timely recognition and appropriate surgical intervention are essential for symptom relief and the prevention of complications.

This case report presents the surgical management of a Grynfelt hernia, in which hernioplasty was performed via a left lumbotomy. The hernia defect was repaired, and its contents were reduced, followed by the placement of a supra-aponeurotic mesh to ensure adequate closure. Due to the rarity of this condition and the lack of specific guidelines in the literature, the surgical approach was determined based on the intraoperative findings.

Surgical techniques for Grynfelt hernia include open repair or minimally invasive approaches such as laparoscopy or robotic-assisted surgery. The choice of approach depends on factors such as the hernia's size, location, and the surgeon's expertise. Further research is needed to clarify management strategies and optimize outcomes for patients with Grynfeltt hernia.

## Figures and Tables

**Table 1:** Classification of lumbar hernias into four types based on six criteria^a^.

Characteristic	A	B	C	D (pseudohernia)
Size (cm)	<5	5–15	>15	—
Location	Superior	Inferior	Diffuse	—
Contents	EP fat	Visceral	Visceral	—
Etiology	Spontaneous	Incisional	Traumatic	—
Muscular atrophy	No (minor)	Mild	Severe	Severe
Recurrence	No	Yes (open)	Yes (laparoscopy)	—
Surgical approach	Open approach EP, TEP laparoscopy	IP laparoscopy	Open approach	Open approach (double mesh)

Abbreviations: EP, extraperitoneal; IP, intraperitoneal; TEP, total extraperitoneal.

^a^The presence of at least two criteria is necessary for defining a type.

## Data Availability

The data that support the findings of this study are available from the corresponding author upon reasonable request.
